# Effects of Foliar Trichomes on the Accumulation of Atmospheric Particulates in *Tillandsia Brachycaulos*

**DOI:** 10.1515/biol-2019-0065

**Published:** 2019-12-31

**Authors:** Ruiwen Zhang, Guiling Zheng, Peng Li

**Affiliations:** 1 College of Resource and Environment, Qingdao Agricultural University, Qingdao 266109, Shandong, China

**Keywords:** air pollution, atmospheric particulate matter, epiphytic plant, foliar trichome, resuspension

## Abstract

Foliar trichomes are specialized structures that have first contact with atmospheric particulate matter (PM), while their effects on PM accumulation are rarely investigated. In this study, epiphytic *Tillandsia brachycaulos* with foliar trichomes was investigated. Trichomes were removed artificially to investigate PM adsorption and resuspension. The results showed that the maximum PM (13.94±0.20 g•m^-2^) and natural PM content (10.65±1.10 g•m^-2^) on *T. brachycaulos* samples with foliar trichomes was significantly higher than those without foliar trichomes. After PM deposition on the leaf surface, more than 85% of PM was dispersed by wind in plants without trichomes, significantly higher than those with trichomes (30.2%). Therefore, the effects of foliar trichomes on PM were reflected not only on PM adsorption, but on PM resuspension. Foliar trichomes can decrease PM resuspension falling on the leaf surface and promote the accumulation of these particles.

## 1 Introduction

With the acceleration of urbanization, the haze problem characterized by atmospheric particles has become a focus of attention. Particulate matter (PM) is a physical pollutant that contains hazardous materials and other elements, causing adverse effects on human health, including a wide range of respiratory and vascular illnesses [1,2]. Plants play a crucial role in the adsorption and reduction of atmospheric particulates [3, 4, 5]. They can accelerate the settlement of particles and effective accumulation of PM, while being eco-friendly with longstanding effects, and in line with public needs [6, 7, 8, 9].

The ability of a leaf blade to accumulate PM is closely related to its surface structure, such as waxes, cuticle, epidermis, stomata and trichomes [10, 11, 12, 13], resulting in differences of 2-3 times the amount of PM among different tree species [14, 15, 16]. Leaf surfaces with high roughness, dense micro-morphological structure and deep depressions have more contact area with particulate matter and, in turn, a higher accumulation of PM [17, 18, 19, 20]. Most studies are based on the comparison of PM among different plants, while investigations on the function of leaf surface structure are limited.

In addition, particles can be temporarily stranded on leaf surfaces, where some will return to the atmospheric environment through resuspension as a result of specific wind or rain events [21, 22, 23, 24]. Although particle resuspension has been studied by a number of researchers [21, 22, 23, 24], particle resuspension following deposition on plant leaves has only been investigated once in a study by Witherspoon and Taylor [25]. They found that *Quercus rubra* L. leaves initially retained more airborne particles than *Pinus strobus* L. However, after an hour of wind activity, the loss of particulate matter on the surface of *Q. rubra* leaves was 90.5% higher than that of *P. strobus* leaves [25]. Therefore, the resuspension of PM on the leaves of plants needs further study to accurately assess the effect of plants on PM.

Epiphytic *Tillandsia* (Bromeliaceae) is an effective air pollution indicator organism since it directly absorbs water and nutrients from the atmosphere. Originating from south and central South America, it is now widely cultivated worldwide [26]. Numerous studies have found that common metals such as Mn, Cu, Fe, Cs, Co, N, Pb and Zn can be detected using the absorption mechanisms of *Tillandsia* [27, 28, 29, 30, 31]. However, epiphytic *Tillandsia* species have not yet been used in monitoring atmospheric particles, even though heavy metals are important components of atmospheric particles.

Foliar trichomes of a distinct morphology have been described in nearly all species of the family Bromeliaceae [26,32]. Trichomes of the *Tillandsia* species are the most important organs for absorption of water and nutrients [26,33,34]. Filhoa et al.[35] found that Hg was highly adsorbed by foliar trichomes but less absorbed by epidermal cells of *Tillandsia usneoides* L. Moreover, specialized trichomes of *Tillandsia velutina* Ehlers facilitate the whole leaf tissue formaldehyde absorption [36]. Nevertheless, information on the role of foliar trichomes of *Tillandsia* in the accumulation of atmospheric particulates is limited. Therefore, *Tillandsia brachycaulos* Schltdl., one common and widespread species in the genus *Tillandsia* but easily distinguished from its varietal twin by its darker green foliage, was selected for study. The following issues were addressed in this study: 1) the function of foliar trichomes of *T. brachycaulos* on PM accumulation, and 2) whether foliar trichomes have an effect on PM resuspension.

## 2 Materials and methods

### 2.1 Materials

*Tillandsia brachycaulos* was bought from the flower market and cultivated in the greenhouse. A total of 120 plants of similar size and healthy growth were randomly divided into four groups, each containing 30 plants. The leaf width and height were measured with a Vernier caliper (accuracy 0.001 mm). Leaf area was calculated as S=AH/2, where A is the width of the leaf base and H is the leaf height, since a leaf of *T. brachycaulos* is triangular in shape.

### 2.2 Artificial removal of foliar trichomes

Foliar trichomes were removed with the adhesive tape method [37,38]. The sticky side of the adhesive tape was lightly pressed onto the adaxial and abaxial surfaces of a leaf five times in order to remove the trichomes. This has proven not to influence the normal absorptive capability of the leaf [39].

All foliar trichomes of group A were removed, while only half the foliar trichomes (determined by leaf area) of group B were removed, and in group C, no foliar trichomes were removed. Group D was the control group, in which trichomes were unremoved, and there was no exposure to PM.

### 2.3 Leaf morphology observation of *T. brachycaulos*

Scanning electron microscopy (SEM) was used to examine leaf surface morphology. Two mature leaves, with and without trichomes, were fixed in FAA stationary liquid (Formalin-Aceto-Alcohol, 70% Alcohol 90 ml, Acetic Acid 5 ml and Formaldehyde 5 ml) for 48 h. The leaves were then dehydrated for 10 min with 30%, 50%, 70%, 80%, 90% and 99.8% ethanol and a mixture of ethanol and tertbutyl (1:1). Finally, the *T. brachycaulos* leaves were soaked with tert-butanol and placed in a refrigerator at minus 20°C to solidify and then freeze dried. Leaves were coated with a 30 nm layer of gold palladium using an ion sputter (KYKY SBC-12) and observed with a scanning microscope (JSF-7500).

### 2.4 Determination of maximum PM content on leaf surface

Leaf maximum PM capturing capability was determined by applying the artificial dust-deposition method. The PM source came from roadside dust and the proportion of particles with different sizes (Table 1) was determined by laser particle size analyzer (Rise2028, Runzhi Technology, China). The plants were perpendicular to the ground. The distance between the artificial dust source and the test plants was 3 m. Particulate matter was not supplied again until the PM fell from the leaves.

**Table 1:** Diameter and ratio of different particulate matters

Diameter (μm)	Ratio (%)	Diameter (μm)	Accumulative ratio (%)
≦1.0	1.52±0.01	≦1.0	1.52±0.01
1.0-2.5	2.49±0.04	≦2.5	4.01±0.05
2.5-5.0	3.18±0.05	≦5.0	7.19±0.03
5.0-10.0	5.23±0.04	≦10.0	12.42±0.05
10.0-50.0	10.33±0.04	≦50.0	22.75±0.06
50.0-100.0	25.47±0.06	≦100.0	48.22±0.09
100.0-300.0	35.19±0.07	≦300.0	83.41±0.09
300.0-500.0	16.59±0.08	≦500.0	100±0

Particulate matter content on the leaf surface was determined by the gravimetric method. After treatments, the plants were immersed in 100 ml of distilled water for approximately 2 h. All leaf surfaces were cleaned using a no-hair-loss paint brush. Then, the leaves were taken out carefully with tweezers and rinsed with distilled water. Each solution was then passed through a pre-weighed (W_1_) glass fiber filter (pore size: 0.45 μm) to collect the PM that was adsorbed on leaf surfaces. The filter papers were then oven dried at 60℃ until the weight was constant (W_2_). The PM capturing capacity for each species was calculated using the equation M=(W_2_-W_1_)/S, where M is PM capturing capacity (g•m^-2^), W_1_ is the weight of filter paper before filtration (g), W_2_ is weight of filter paper after filtration (g), and S is the leaf area of the plant (m^2^).

### 2.5 Determination of PM resuspension content on the leaf surface

The plants were placed in an indoor environment without wind until reaching maximum PM. The plant weight at maximum PM accumulation (W_max_) was used to determine the PM resuspension amount, based on which the resuspension ratio was calculated. According to local common wind **s**peed, three different wind speeds (4.8 m•s^-1^, 5.4 m•s^-1^ and 6.0 m•s^-1^) were set, which were provided by a fan and determined by a wind velocity indicator (Cole-Parmer, USA), to measure the effect of wind speed on PM resuspension. Three operating times of the wind (10 min, 20 min and 30 min) at wind speed 4.8 m•s^-1^ were also set to measure the effect of operating time on PM resuspension.

When the set wind blowing time was reached, the plant weight at this time (W_3_) was determined, and the ratio of PM resuspension on leaf surfaces was calculated using the following equation: R=(W_max_-W_3_)/ W_max_*100%, where R is the ratio of PM resuspension (%), W_max_ is maximum PM content on leaf surface (g), and W_3_ is plant weight after wind action (g).

### 2.6 Determination of natural PM content on leaf surface

Samples of *T. brachycaulos* were washed with deionized water, air-dried and transferred to web plastic baskets containing wet wood to protect them against excessive hydropenia. The baskets were then hung outside at a height of 3 m on top of containers (20 ft, dry standard). The samples were exposed to air for 15 days, and the natural PM content on the leaf surface was determined by using a gravimetric method, a method similar to determining maximum PM content. The control group (D) was placed in an indoor semi open seal, and the plants were exposed to a similar amount of sunlight and moisture.

### 2.7 Data analysis

Statistical analysis of the data was performed using SPSS 19.0 (IBM, USA). Normal distribution of the data was first checked based on Kolmogorov-Smirnov (K-S) index. One-way analysis of variance (ANOVA) was calculated to determine if significant differences occurred among different treatments. The least significant difference (LSD) multiple comparisons tests were performed to determine which treatment exhibited significant differences, where *P*<0.05. Graphs were constructed using Excel 2003.

## 3 Results

### 3.1 Leaf surface morphology of *T. brachycaulos*

**Fig. 1:**
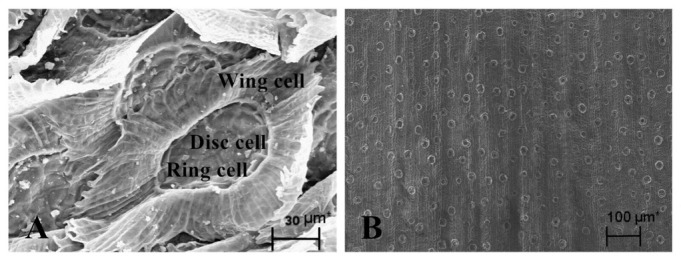
Leave surface of *T. brachycaulos* under SEM. A, Foliar trichomes of *T. brachycaulos*; note PM on the trichomes; B, Leaf surface after trichomes removal

The leaf surface of *T. brachycaulos* is covered with dense foliar trichomes (Fig. 1A). The trichomes consist of three layers of cells. The centre is four disc cells, which are surrounded by 8 ring cells. The outer layer has irregular long wing cells, which are closely connected with each other and have many short transverse stripes. Following artificial removal of foliar trichomes, almost all wing cells were removed, and the epidermal cells and stomata that were originally covered by trichomes were now exposed (Fig. 1B).

### 3.2 Relationship between foliar trichomes and maximum PM content in T. brachycaulos

**Fig. 2:**
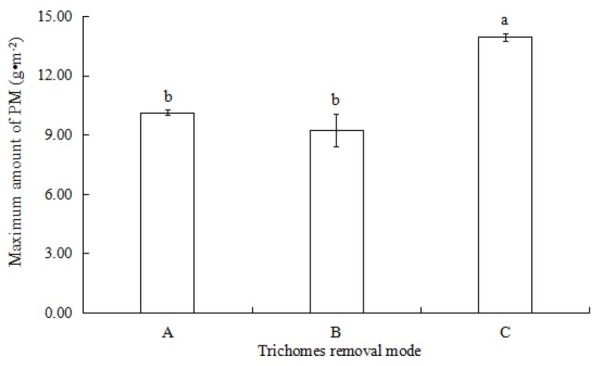
The maximum amount of PM in *T. brachycaulos* Notes: A, Without foliar trichomes; B, With 1/2 trichomes; C, With foliar trichomes

In cases where foliar trichomes were not removed, the maximum amount of PM per unit leaf area was 13.94±0.20 g•m^-2^ (Fig. 2), which was significantly higher (one-way ANOVA, *P*<0.05) than that without trichomes (10.12±0.13 g•m^-2^). The maximum amount of PM on the leaf surface with half the trichomes removed was 9.23±0.82 g•m^-2^, which had no significant difference compared to that with all trichomes (*P*>0.05).

### 3.3 Relationship between foliar trichomes and natural PM content

**Fig. 3:**
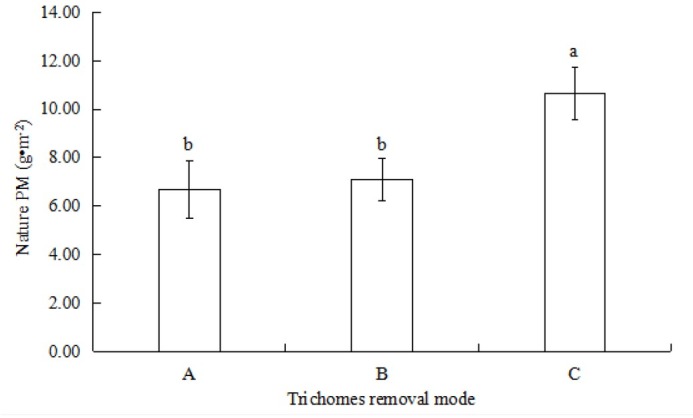
The natural amount of PM in *T. brachycaulos*. Notes: A, Without foliar trichomes; B, With 1/2 trichomes; C, With foliar trichomes

The amount of natural PM on the leaf surface of *T. brachycaulos* (Fig. 3) was lower than the maximum amount of PM (Fig. 2). However, the effects of foliar trichomes on the natural PM deposition were similar to that of the maximum PM adsorption. The amount of natural PM in the group with foliar trichomes (10.65±1.10 g•m^-2^) was almost twice as high as that without trichomes (6.70±1.19 g•m^-2^). There was no significant difference between the two groups with all trichomes and with half trichomes (7.09±0.87 g•m^-2^).

### 3.4 Relationship between foliar trichomes and PM resuspension

#### 3.4.1 Effect of wind speed

**Fig. 4:**
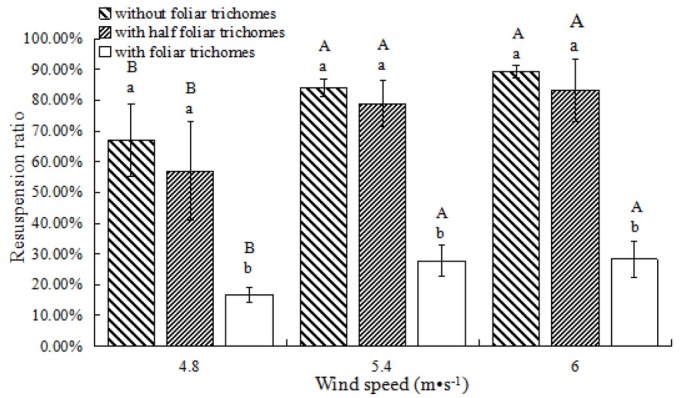
Resuspension ratio of PM in *T. brachycaulos* after 10 min at different wind speeds. Notes: Different small letters mean significant difference at 0.05 level among different treatments of foliar trichomes after the same time, and different capital letters mean significant difference at 0.05 level among different time for the same treatment of foliar trichomes.

When wind speeds increased from 4.8 m•s^-1^ to 5.4 m•s^-1^, the PM resuspension ratio increased significantly for plants with and without foliar trichomes (Fig. 4, *P*<0.05). However, there was no significant difference in the PM resuspension ratio at wind speeds of 5.4 m•s^-1^ and 6.0 m•s^-1^ (*P*>0.05).

The more foliar trichomes were removed on the leaves of *T. brachycaulos*, the higher the observed PM resuspension ratio (Fig. 4). The PM resuspension ratio for plants with trichomes was significantly lower than those without trichomes or with half trichomes (*P*<0.05), while the latter two groups showed no significant differences (*P*>0.05), irrespective of wind speed.

#### 3.4.2 Effect of operating time of wind

**Fig. 5:**
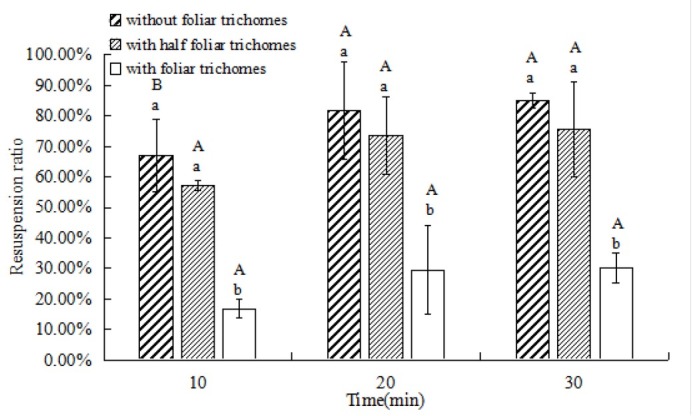
Resuspension ratio of PM in *T. brachycaulos* at 4.8 m•s^-1^ wind speed at different times. Notes: Different small letters mean significant difference at 0.05 level among different treatments of foliar trichomes after the same time, and different capital letters mean significant difference at 0.05 level among different time for the same treatment of foliar trichomes.

When the time increased from 10 min to 20 min, the PM resuspension ratio increased significantly for plants without foliar trichomes (Fig. 5, *P*<0.05). However, there was no significant difference in the PM resuspension ratio at time of 20 min to 30 min.

The PM resuspension ratio for plants with trichomes was significantly lower than those without trichomes or with half trichomes (*P*<0.05), while the latter two had no significant difference (*P*>0.05). When the wind time reached 30 minutes, PM resuspension ratios were 85.2%, 75.6% and 30.2% for those plants with no trichomes, half trichomes and with trichomes, respectively.

## 4 Discussion

Leaf surface structure and cell morphology have a large influence on the effects of plant dust accumulation [40, 41, 42]. Generally, plants with surface roughness favour the adsorption of PM [43,44]. The highest amount of PM removed by *Mangifera indica* L. with deep grooves and high stomata density was 12.72 g•m^-2^ in an industrial area; this was significantly higher than *Bauhinia blakeana* Dunn. (1.48 g•m^-2^), which possesses stomata that are regularly arranged [45]. Similarly, the results of our experiment showed that both the maximum PM amount and natural PM of *T. brachycaulos* with trichomes were significantly higher than plants without trichomes (Fig. 2, 3).

Foliar trichomes in *Tillandsia* may play a more significant role in the accumulation of atmospheric PM than other leaf appendages. The maximum PM amount of *T. brachycaulos* with trichomes was 13.94 g•m^-2^ (Fig. 2), higher than those of *Plum* and *Honeysuckle*, which have downy leaves (11.5 g•m^-2^ and 10.8 g•m^-2^, respectively) [11]. For those plants with leaf surface appendages, foliar trichomes are the structures first exposed to atmospheric particles, as opposed to leaf epidermal cells. Their distribution density, morphology, texture and type may directly affect the process of PM adsorption and resuspension on the leaf surface. Foliar trichomes of *Tillandsia* are formed by three cell types: wing cells, ring cells and disc cells (Fig. 1). The disc cells are connected with mesophyll cells through the stem cells [26]. When absorbing moisture and nutrients, the wing cells capture the water and nutrients from the air and then transfer them from ring cells, disc cells and stem cells to mesophyll cells, based on the “syphonage” principle [26,46,47]. Theoretically, long wing cells of *Tillandsia* are suitable for the adsorption of atmospheric PM when they are capturing moisture and nutrients. Moreover, foliar trichomes densely cover the leaf surface of *T. brachycaulos* (Fig. 1A), which can further promote the accumulation of atmospheric PM.

Nevertheless, there was no significant difference in PM content between the two groups, those without trichomes and those with half trichomes of *T. brachycaulos* (Fig. 2, 3). This suggests that the accumulation of PM was not proportional to the number of foliar trichomes, and the trichome was not the only factor influencing PM accumulation. Other leaf features such as leaf shape, roughness, leaf inclination, epidermal cells and epicuticular waxes can also affect the accumulation of atmospheric PM [8,19,48, 49]. When the foliar trichomes were artificially removed, only the wing cells of the *Tillandsia* were removed; the other two cells were still present on the leaf surface (Fig. 1). The adsorption function of foliar trichomes after they are removed does not disappear but only becomes weaker.

In addition, atmospheric PM temporarily lands on the leaf surface, and some particles return to the atmosphere due to wind or rain, leading to resuspension. Whilst Ould-Dada and Baghini [50] believe that wind speed of less than 5 m•s^-1^ does not affect the amount of PM adsorbed by plant leaves, most studies show that wind plays an important role in the resuspension of atmospheric PM. For example, more than 80% of particles are blown up from *Firmiana platanifolia* Marsili. and as much as 90% from *Magnolia liliflora* Desr. [11]. The adsorption capacity of PM is lower in strong wind conditions than in calmer conditions [3]. Regardless of the increase in wind speed or the extension of wind action time, the proportion of PM resuspension increased significantly in our experiment. Moreover, our study showed that by increasing removal of foliar trichomes of *T. brachycaulos*, particle resuspension increased (Fig. 4, 5). Thus, effects of foliar trichomes on the adsorption and resuspension of atmospheric particulates was reflected, not only in the total amount of atmospheric particulates, but also in the process of atmospheric particulate resuspension. Foliar trichomes can decrease the resuspension of atmospheric particles falling on the leaf surface and promote the adsorption of these particles.
